# Close to Home: Evidence on the Impact of Community-Based Girl Groups

**DOI:** 10.9745/GHSP-D-20-00015

**Published:** 2020-06-30

**Authors:** Miriam Temin, Craig J. Heck

**Affiliations:** a Poverty, Gender, and Youth Research Program, Population Council, New York.

## Abstract

**Purpose:**

Community-based programming to promote gender equity, often delivered through community-based girl groups (CBGGs, sometimes called “safe spaces”), is increasing. However, evidence is weak on how CBGGs are implemented and their effect on adolescent girls’ health and well-being. We conducted a comprehensive literature review to identify relevant CBGG programs.

**Methods:**

The review included programs with impact evaluations that used experimental or quasi-experimental design, data from 2 time points, control/comparison groups, and quantitative program effects and *P* values.

**Results:**

We analyzed evaluations of 30 programs (14 randomized controlled trials, 16 quasi-experimental). Although program designs varied, most programs targeted unmarried girls aged 13 to 18 years who were both in school and not in school, and who met weekly in groups of 15 to 25 girls. Nearly all programs used multisectoral approaches focusing on life skills and often economic and financial content, such as financial literacy and microsavings. Complementary activities with community members, boys, and health services were common. Across programs, evaluations reported statistically significant effects (*P*<.05) the majority (>50%) of times they measured outcomes related to gender and health attitudes and knowledge, education, psychosocial well-being, and economic and financial outcomes. Measures of outcomes related to girls’ health behaviors and health status had majority null findings.

**Conclusions:**

CBGG program evaluations found positive effects on girl-level outcomes that are independent of external factors, like gender norm attitudes, and suboptimal performance on health behavior and health status, which rely on other people and systems. This delivery model has promise for building girls’ assets. Complementary actions to engage girls’ social environments and structures are needed to change behaviors and health status.

## Abstract (precis)

Available evidence, though limited, shows that programs can use community-based girl groups to help adolescent girls improve attitudes toward gender roles and norms, early pregnancy, and child marriage; evaluations indicate they have suboptimal performance on health behavior and health status.

## Teaser

**Key Findings:**

- Evaluations of community-based girl groups (CBGGs) programs—sometimes called safe spaces—reported positive effects on girl-level outcomes that are independent of external factors and suboptimal performance on health behavior and health status.
- The limited evidence available shows that CBGGs have the potential to contribute to adolescent girls’ empowerment; complementary activities are needed to mitigate risk.

**Key Implications:**

- Program implementers should consider the role of female mentor-led girl groups in improving adolescent girls’ attitudes, beliefs, knowledge, and awareness on health and gender.
- Policy makers and funders should recognize that to change behaviors and sustainably reduce risk, CBGGs should be combined with action to engage girls’ social environments and structures.
- Researchers should conduct rigorous implementation science and impact evaluation studies of CBGGs to learn more about effective practices and the likely impact of CBGGs for vulnerable subpopulations of adolescent girls.

## INTRODUCTION

Governments in countries that have populations of median age under 25^1^ face demographic pressure as the result of infant mortality gains and high birth rates. Their young age structures offer an unprecedented opportunity for progress, which has stimulated global commitment to adolescents and, in particular, adolescent girls. Although attention to adolescent girls in low- and middle-income countries (LMICs) has increased dramatically,^2^^,^^3^ hundreds of millions of adolescent girls still lack access to essential services and basic human rights. Despite progress, globally 12 million girls are still married as children annually,^4^ and in sub-Saharan Africa, 35% of girls—versus 30% of boys—are not in school.^5^

Girls at the highest risk of the worst outcomes—like child marriage, early pregnancy, and HIV infection—often miss the benefits of social sector programs because of their socially isolated and marginalized status. Girls who lack contact with schools, where youth programs often take place, also may be excluded from formal health and financial services and labor markets. Adolescent girls with access to health facilities rarely receive adolescent-friendly services; providers may overlook their specific health needs or treat them insensitively.^6^

Girls at the highest risk of the worst health outcomes often miss the benefits of social sector program because of social isolation and marginalization.

Some programs use community-based girl groups (CBGG) to address risk for girls who are hard to reach through formal delivery channels like schools and health services. In CBGG programs, girls and young women meet regularly with a leader (e.g., a mentor) who uses a variety of pedagogical methods to address sexual and reproductive health (SRH), HIV prevention, life skills, economic and financial outcomes, and other topics.

CBGGs are proliferating across geographic regions. For example, under the Determined, Resilient, Empowered, AIDS-free, Mentored, and Safe (DREAMS) Partnership to reduce HIV infections among adolescent girls and young women, implementing partners in 14 countries in sub-Saharan Africa and Haiti use CBGGs to build adolescent girls’ and young women’s social and other assets (e.g., cognitive, economic, health assets).^7^ Often, these are called “safe space” programs because they meet in community-based venues that girls and parents perceive as safe and private, which can reduce barriers to attendance and enable discussion of sensitive issues. The Population Council tests the CBGG model based on a theory of change that posits when multisectoral programs address girls holistically, content is tailored to respond to heterogeneous girl segments, and group meetings are accessible and mentor-led, they can build girls’ protective assets and empower them to reduce risk and increase opportunity in the right environment.^8^

Increasingly, randomized controlled trial (RCT) evidence joins the body of quasi-experimental studies of CBGG programs, expanding both the amount and type of evidence available. However, this evidence is not always available to funders and implementers in an accessible form they can use to inform decision making. One explanation is there has been little analysis of the evaluation evidence specific to CBGG programs, although they are included in broader reviews.^9^^–^^11^ The time is right to consolidate what is known about CBGGs to help donors, researchers, policy makers, and implementers make informed decisions regarding funding, research, policy, and practice.^12^

To help fill the gap between evidence generation and evidence use, we conducted the first-ever literature review focused on the evidence on CBGG programs. We explored how programs with CBGGs were designed and their effects. We also identified questions that merit further research to inform programming to empower girls and advance their well-being. By critically reviewing impact evaluation evidence on CBGGs in LMICs, we aimed to answer 4 questions:

1. What design features do CBGGs with impact evaluations have?
2. What did those evaluations measure?
3. What were the program effects on girls?
4. What type of study designs generated which results?

We conducted the first-ever literature review dedicated to CBGG program evidence.

The literature on CBGG programs was subjected to rigorous selection, search, abstraction, and analysis methods to produce a holistic, informed assessment of this program delivery model.

## METHODS

### Study Selection

We reviewed literature in search of evaluations of programs that used group-based methods to deliver content to adolescent girls to build their life skills and empower them. To be considered for our analysis, the program had to include: (1) a group of 10- to 19-year-old girls who met regularly (i.e., more than once); (2) a female mentor who received dedicated training for the role; and (3) a meeting venue located in a community setting rather than a formal institution (e.g., not hospitals or schools during formal classroom hours). We considered group leaders as “mentors” if they were at least slightly older than participants, consistent with the majority of programs in our sample; peer educators also were considered if they fit our criteria.

Programs underwent 2 levels of screening to be included in our analysis. The first screening assessed if the evaluated program included the elements described above. The second screening focused on the rigor of the evaluation methodology. To clear this screening, study designs had to have: an impact evaluation that used an experimental or quasi-experimental study design, data collected at a minimum of 2 time points, an intervention and control/comparison group, and quantitative program effects and probability values (*P* values). We also included descriptive publications (i.e., those that did not report *P* values) if they provided supporting information about programs that were described in other papers in our sample. Evaluations that constructed a post hoc comparison group using statistical methods, such as propensity score matching, did not pass this screening.

We limited our search to peer-reviewed and non-peer-reviewed (gray) literature in English published between 2000 and 2017.

### Participants

In our review, we sought evaluations of programs that targeted adolescent girls aged 10 to 19 years who were married or unmarried. Programs with young women (i.e., aged 20–24 years) were included only if adolescent girls also were enrolled. For programs with older participants (i.e., aged over 24 years), the analyses had to be stratified by or controlled for age to pass our screening. Programs that included adolescent boys and young men also passed the screening if their analyses controlled for sex or disaggregated results.

### Outcomes

To understand the programs’ operations and reported effects, we assessed both implementation science and impact evaluation findings. The evaluations used a large variety of impact measures across programs that encompassed both proximal and distal effects on outcomes. Program evaluations relied heavily on self-reported data, and a few used objective methods to measure the effects (e.g., biomarker testing for HIV, herpes simplex virus 2 [HSV-2], pregnancy status; banking information about savings amounts; problem sets to gauge numeracy and literacy levels).

### Search Strategy

We searched for related publications and captured them based on a review of titles, abstracts, and summaries. To identify papers for our sample, we consulted systematic and other reviews of evidence on interventions for adolescents^11^^,^^13^^–^^16^ and 3ie’s evidence gap map on adolescent SRH.^17^ We also consulted research and journal databases (e.g., Google Scholar, JSTOR, EBSCO’S Academic Search Complete, POPLINE, and DeepDyve) using key words including “girl-centered,” “safe spaces,” and “mentor.” We also reviewed web sites of relevant implementing organizations with a history of programming for adolescent girls in LMICs. Programs outside LMICs were excluded.

### Data Extraction

We extracted program details including: design features (country, setting); program aims; descriptions of participant details (girls’ characteristics, mentor qualifications); group characteristics (group size, meeting frequency, program duration, topics covered including health services and male engagement activities); and evaluation details (sample size, program effects).

### Data Analysis and Synthesis

For reporting purposes, we created and defined effect categories based on the description in the evaluations and the stated program goals. To enable the interpretation of the wide range of evaluation results, we constructed 8 outcome domains that aggregated the range of effects evaluated. The outcome domains are: (1) health beliefs and attitudes, (2) gender beliefs and attitudes, (3) education-related outcomes, (4) psychosocial outcomes, (5) health and gender knowledge and awareness (6 of 7 on health), (6) economic and financial outcomes, (7) health-related behavior, and (8) health status. If evaluations used multiple indicators to assess the same outcome, we combined them into 1 aggregated effect per study. For example, in the psychosocial outcome domain, social support is a composite of numerous indicators: sociability, number of friends, ability to go to girl/youth groups, has at least 1 social safety net, social inclusion index, and others (Table 1).

**TABLE 1.** Community-Based Girl Group Program Effects by Outcome Domains

	**Reported Effect Measure(s):Beneficial Out of Total** ^a^
Health beliefs/attitudes	
Improved attitudes toward early pregnancy	2/2
Increased concerns about unprotected sex	2/2
Increased demand for health services^b^	2/2
Affected their perceived vulnerability to HIV/AIDS	1/1
Improved attitudes toward female genital mutilation/cutting	1/1
Improved attitudes toward family sizes	2/3
	10/11 (90.9%)
Gender beliefs/attitudes	
Changed perception of gender roles and norms	7/8
Improved attitudes towards child marriage	5/6
Improved attitudes towards gender-based violence	4/7
Improved beliefs regarding girls' education	1/2
Improved attitudes towards girls’ economic empowerment	1/2
	18/25 (72.0%)
Education-related outcomes	
Improved numeracy skills	4/4
Increased vocational training	1/1
Reduced need for tutoring	1/1
Increased school enrollment	3/4
Improved literacy skills	2/4
Increased school retention	1/3
Increased grade attainment	1/3
	13/20 (65.0%)
Psychosocial outcomes	
Increased self-efficacy regarding condom use	2/2
Increased self-efficacy to assert opinions and concerns	6/7
Increased social support^c^	7/9
Increased self-efficacy to seek out HIV testing	1/1
Increased autonomy when searching for a job	2/3
Increased mobility	4/10
Improved self-esteem	1/3
Reduced experience of gender discrimination	0/1
	23/36 (63.8%)
Knowledge/awareness-health	
Increased HIV knowledge	9/12
Increased reproductive health knowledge	6/10
Increased STI knowledge	5/9
Increased menstrual regulation knowledge	1/2
Increased awareness of sexual and reproductive health and HIV	1/2
Knowledge/awareness-gender	
Increased awareness of marital-related rights^d^	2/4
	24/39 (61.5%)
Economic and financial outcomes	
Increased household assets	1/1
Decreased food insecurity	1/1
Increased monthly expenditures	1/1
Increased number of savings accounts (formal and informal)	7/8
Increased employment	5/6
Increased earnings	2/4
Increased savings amount	1/4
Increased financial literacy	0/3
Reduced dowry practices	0/2
	18/30 (60.0%)
Health-related behavior	
Increased secondary abstinence	1/1
Increased menstrual hygiene management	1/1
Increased utilization of violence treatment, support, and/or prevention services	1/1
Increased health service utilization	3/6
Reduced child marriage	3/8
Increased condom use	5/11
Increased contraceptive use	3/9
Delayed sexual debut	2/6
Decreased transactional sex	1/3
Decreased number of sex partners	1/7
Reduced drugs or alcohol misuse	0/1
Increased HIV testing	0/2
	21/56 (37.5%)
Health status	
Improved self-rated health status	1/1
Decreased female genital mutilation/cutting	1/1
Decreased pregnancies^e^	2/5
Decreased experience of physical violence	1/3
Decreased experience of sexual violence^f^	2/7
Decreased HSV-2 incidence	1/4
Delayed pregnancy	0/1
Decreased negative mental health outcomes	0/2
Reduced STI symptoms	0/3
Decreased HIV incidence	0/4
	8/31 (25.8%)

Abbreviations: HSV-2, herpes simplex virus 2; STI, sexually transmitted infection.

a Denominator=total number of times each effect was measured across all the programs. Numerator=number of times a measured effect was statistically significant (i.e., beneficial). A program could not contribute to an effect’s denominator and numerator more than once. An aggregate total of the effect measures is presented at the bottom of each domain.

b For example, contraceptives and voluntary counseling and testing.

c Social support includes increasing girls’ support from elders (e.g., family and non-family adults whom girls can turn to in need) and peers (e.g., social safety nets, girls’ clubs, friends).

d For example, detriments of child marriage and legal age of marriage.

e This effect includes early, unintended, and current pregnancies.

f Aggregates the measurement of indicators that described experiences of rape and indecent/unwanted touching by someone of the opposite sex, including a husband.

Within each domain, we report beneficial—statistically significant (α=0.05) changes in the intended direction (i.e., protective direction [<null value] for detrimental outcomes and positive direction [>null value] for advantageous outcomes)—and null (nonsignificant) measures for each effect. We also assessed the total number of times that evaluations measured effects in each outcome domain across the programs. Analyzing effect sizes was beyond the scope of the review. We considered unintended effects as a statistically significant change in the detrimental direction but excluded them from the analysis.

### Ethics

Since this study did not involve human subjects research, we did not seek institutional review board approval.

## RESULTS

### Literature Search Results

**FIGURE 1.**
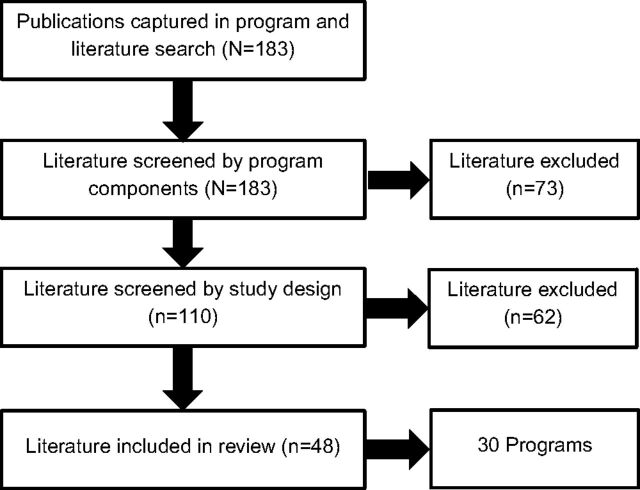
Results of Literature Search on Community-Based Girl Group Program Evaluations

The initial review produced 183 manuscripts, articles, and reports. The first screening eliminated 73 documents; we subjected the remaining 110 publications to the second screening and removed an additional 62 whose evaluation design did not meet our requirements. This left 48 publications that reported on evaluations of 30 programs: 14 RCTs and 16 using quasi-experimental design (Figure 1). The program details and reported findings for these programs are found in Table 2. Sixty percent of programs took place in Africa (Eastern: 40%, Southern: 20%), and one-third occurred in South/Southeast Asia.

**TABLE 2.** Details of Final Sample of Community-Based Girl Group Programs, N=30

**Program Name, Date**	**Country and Setting**	**Program Design and Aims**	**Participant Details**	**Group Characteristics and Content**	**Evaluation Details and Program Effects** ^a^
The Bangladeshi Association for Life Skills, Income, and Knowledge for Adolescents (BALIKA),^18^ 2013–2015	Bangladesh, rural	Randomized control trial Aims: Delay marriage among adolescent girls by offering skills-building approaches aimed at empowering girls in 3 Bangladesh communities with highest child marriage rates: Khulna, Satkhira, and Narail	Girls: 12–18 years old, in and out of school, unmarried Mentors: Local, young, slightly older than participants	Met weekly, 2–3 hours, 18 months’ duration Topics: Education arm: math and English tutoring (in-school girls), computing or financial training (out-of-school girls) Gender-rights arm: Life skills training on gender rights and negotiation, critical thinking, and decision making Livelihoods skills training arm: Training in computers, entrepreneurship, mobile phone servicing, photography, and basic first aid All arms: Community engagement activities, basic life skills, exposure to using computers and tablets	Sample size: 7,452 intervention (2,516 education arm, 2,460 gender awareness arm, 2,476 livelihoods arm), 2,530 control/comparison Effects: *Increased health service utilization* *Increased menstrual hygiene management* *Reduced child marriage* *Improved numeracy skills* *Increased school retention* *Increased school enrollment* *Reduced need for tutoring* *Increased social support* *Increased employment* *Increased HIV knowledge* *Increased RH knowledge* *Increased STI knowledge* *Improved attitudes toward child marriage* *Improved attitudes toward GBV* Reduced dowry payments Increased contraceptive use Increased mobility Reduced experience of gender discrimination Increased menstrual regulation knowledge Increased awareness of marital-related rights Changed perception of gender roles and norms
Empowerment and Livelihoods for Adolescents (ELA): Bangladesh,^19^ 2005–2007	Bangladesh, rural	Quasi-experimental Aims: Assess program’s usefulness in terms of delaying age of marriage, keeping girls enrolled in school, enhancing sociability, and increasing mobility and awareness about health issues	Girls: 10–24 years old, in and out of school, married and unmarried Mentors: BRAC program supervisor	30 girls, met weekly, 2–3 hours Topics: Health, life skills training, microfinance, girls' rights, books, games	Sample size: 322 intervention, 242 control/ comparison Effects: *Increased mobility* *Increased social support* *Increased earnings* Increased savings amount Increased financial literacy
Growing Up Safe & Healthy,^20^ 2012–2013	Bangladesh, urban	Randomized control trial Aims: Improve sexual and RH and rights, reduce intimate-partner violence among women and girls in urban slums, reduce child marriage	Girls: 10–35 years old, in and out of school, married (15–29-years old) and unmarried (10–14 years old) Mentors: Observed leadership qualities, rapport with community, willingness to work on campaign activities	15 girls, 20 months’ duration Topics: Life skills training, legal rights/GBV, referrals to health or legal services Also included: Boys/young men engagement	Sample size: 2,656 intervention^b^ (1,910 female [15-19 years old], 746 male [18–24 years old]),1287 control/comparison^b^ (952 female [15–19 years old], 335 male [18–24 years old]) Effects: *Decreased experience of physical violence* *Decreased experience of sexual violence* Reduced child marriage
Kishori Abhijan,^21^ 2001–2003	Bangladesh, rural	Quasi-experimental Aims: Promote a gender-equitable environment where girls can broaden their choices, participate in empowering social and economic processes, and realize their potential as agents for social change	Girls: 10–19 years old, in and out of school, married and unmarried Mentors: Employed at BRAC or Center for Mass Education and Science, demonstrated experience and capacity working with adolescent girls	Group characteristics information was not specified Topics: Life skills training, legal rights, gender, economic empowerment (savings accounts, credit access)	Sample size: 1,901 intervention, 310 control/ comparison Effects: *Increased employment* Reduced child marriage Increased school retention Reduced dowry practices
Ishraq,^22^^–^^25^ 2001–2013	Egypt, rural	Quasi-experimental Aims: Create safe spaces where out-of- school girls can learn, play, and build self-confidence, improve out- of-school girls' knowledge and attitudes regarding transitions to adulthood (e.g., early marriage, RH, and education)	Girls: 13–15 years old (pilot), 11–15 years old (scale-up), out of school (both phases) Mentors: Local, at least secondary school education	30 girls, met 4 days/week, 4 hours, pilot for 30 months, scale-up for 20 months duration Topics: Life skills training, sports, livelihoods training, domestic skills, legal rights, IDs/ official documentation, financial education, nutrition Also included: Boys/young men engagement	Sample size: Pilot: 453 intervention, 134 control/comparison Scale-up: 1,321 intervention, 539 control/comparison Effects: *Decreased female genital* *mutilation/cutting*^c^ *Improved numeracy skills Improved* *literacy skills* *Increased self-efficacy to assert opinions* *and concerns* *Increased RH knowledge* *Improved attitudes toward child marriage* *Improved attitudes toward family sizes* *Improved attitudes toward female genital* *mutilation/cutting* *Changed perception of gender roles and* *norms* Increased health service utilization Increased mobility Improved self-esteem Improved attitudes toward GBV Improved beliefs regarding girls' education
Berhane Hewan,^26^ 2004–2006	Ethiopia, rural	Quasi-experimental Aims: Improve educational attainment, RH knowledge, contraceptive use, and age at first marriage	Girls: 10–19 years old, in and out of school, married and unmarried Mentors: 10th grade education	15–20 girls, unmarried girls met 5 days/week, married girls met weekly Topics: Nonformal education, livelihoods training, referrals to RH services	Sample size: 650 intervention^b^, 736 control/ comparison^b^ Effects: *Increased contraceptive use* *Reduced child marriage* *Increased school enrollment* *Increased HIV knowledge* Increased awareness of sexual and RH *and HIV/AIDS* Increased STI knowledge Improved literacy skills Increased grade attainment
Biruh Tesfa,^27^^–^^29^ 2006–2016	Ethiopia, urban	Quasi-experimental Aims: Increase social networks and support to poorest, most marginalized girls in poorest urban areas of Ethiopia; improve girls’ knowledge and skills to prevent HIV	Girls: 7–18 years old, out of school, married and unmarried Mentors: Adult women from the community	Met 5 days/week, 2 hours, 38 sessions Topics: Life skills, HIV counseling and treatment, financial literacy, vouchers for health care, school materials	Sample size: Gondar: 767 intervention,^b^ 405 control/ comparison^b^ Addis Ababa: 630 intervention, 646 control/comparison Effects: *Increased health service utilization* *Improved numeracy skills* *Improved literacy skills* *Increased school enrollment* *Increased social support* *Increased HIV knowledge* *Increased demand for health services* Increased HIV testing Increased grade attainment
Better Life Options,^30^ 2006–2008	India, rural	Quasi-experimental Aims: Enhance girls' awareness of sexual and RH matters; build agency in terms of mobility, decision making, and sense of self-worth; foster egalitarian gender role attitudes; develop vocational skills and future work aspirations; influence perceptions about marriage and their ability to negotiate marriage- related decisions and success in delaying marriage and first pregnancy	Girls: 13–17 years old, in and out of school, unmarried Mentors: Young, educated, articulate, local, can manage big groups	15–20 girls, met almost daily, 2 hours, 6–9 months’ duration Topics: Life skills training, livelihoods, sports	Sample size: 810 intervention, 228 control Effects: *Increased mobility* *Increased number of savings accounts* *(formal & informal)* *Increased HIV knowledge* *Increased STI knowledge* *Increased awareness of marital-related* *rights* *Improved attitudes toward child marriage* *Changed perception of gender roles and* *norms* Reduced child marriage Increased self-efficacy to assert opinions and concerns Increased RH knowledge Increased awareness of sexual and RH and HIV
First-time Parents Project,^31^ 2003–2004	India, rural	Quasi-experimental Aims: Develop and test integrated package of health and social interventions to improve married young women's reproductive and sexual health knowledge and practices, enhance their ability to act in their own interest, and expand their social support networks	Girls: Mean age 19.4 years old, in- school status not reported, only years of schooling completed, married Mentors: Staff of Child In Need Institute or Deepak Charitable Trust	8–12 girls, met monthly, 2–3 hours Topics: Legal literacy, vocational training, savings and credit management, pregnancy, gender, spousal relationships Also included: Access and quality improvements of health services	Sample size: Diamond Harbour: 403 intervention, 259 control Effects: *Increased self-efficacy to assert opinions* *and concerns* *Increased social support* *Increased STI knowledge* *Changed perception of gender roles and* *norms* Increased contraceptive use Increased mobility Improved attitudes toward GBV
Promoting Change in Reproductive Behavior in Bihar (PRACHAR),^32^^–^^36^ 2001–2004	India, rural	Quasi-experimental Aims: Change beliefs of people 12–24 years old about RH/FP, challenge traditional behavior patterns of early childbearing and inadequate spacing between children, and promote informed and healthy reproductive behavior; change parents’ beliefs and influential community adults about RH/FP, provide knowledge to discourage early marriage of daughters, curb pressure on young couples for early childbearing, and encourage adequate spacing of subsequent children; increase use of contraceptives among young married couples, particularly to delay first child until mother is mature, and to space subsequent births by at least 3–5 years	Girls: 15–24 years old, in and out of school, married and unmarried Mentors: Semi-literate, known and respected by community members	30 girls, Phase 1 duration: 21 months (Patna) 24 months (Nawada) 27 months (Nalanda) Phase 2 duration: Not specified Phase 3 duration: 7 months Topics: Sexual and RH, nutrition, spousal negotiation, gender norms Also included: Boys/young men engagement, access and quality improvements of health services	Sample size: Phase 3: 2,171 intervention (1,382 female, 789 male), 1,050 control/ comparison (679 female, 371 male) Effects: *Increased contraceptive use* *Increased grade attainment* *Increased mobility* *Increased self-efficacy to assert opinions* *and concerns* *Increased autonomy when searching for a* *job* *Increased number of savings accounts* *(formal & informal)* *Increased HIV knowledge* *Increased RH knowledge* *Increased menstrual regulation knowledge* *Increased awareness of marital-related* *rights* *Improved attitudes toward child marriage* *Improved attitudes toward early pregnancy* *Increased demand for health services* *Changed perceptions of gender roles and* *norms* Reduced child marriage Delayed pregnancy
Improving Learning Outcomes and Transition to Secondary School Study,^37^ 2013–2015	Kenya, urban	Quasi-experimental Aims: Promote access to and improve the quality of secondary education among girls who live in informal urban settlements	Girls: 12–19 years old, in school Mentors: 21–40 years old, completed secondary school	230 after-school sessions, 34 life skills sessions Topics: Life skills training, homework support on numeracy and literacy	Sample size: 855 intervention, 416 control/comparison Effects: *Improved numeracy skills* Improved literacy skills
Nyeri Youth Health Project,^38^ 1998–2000	Kenya, urban and rural	Quasi-experimental Aims: Delay sexual debut among sexually inexperienced youth, prevent negative sexual health outcomes among sexually experienced youth, create RH information and service environment that was responsive to information and service needs of young people	Girls: 10–24 years old, in and out of school, unmarried Mentors: Local, respected, well-known adults and young parents	Met weekly, 90–120 minutes, 4–8 weeks’ duration Topics: Life skills training	Sample size: 2,504 intervention^b^ (1,220 female, 1,284 male),905 control^b^ (472 female, 443 male) Effects: *Decreased number of sex partners* *Increased secondary abstinence* *Increased self-efficacy to assert opinions and* *concerns* Increased condom use Delayed sexual debut
Safe and Smart Savings,^39^ 2008–2010	Kenya, urban	Quasi-experimental Aims: Develop, pilot test, and roll-out individual savings accounts to girls belonging to girls' groups Program evaluation aims: Understand the social, economic, and health effects of participating in program activities	Girls: 10–19 years old, in and out of school, unmarried Mentors: Young women from community	15–25 girls, met weekly, 30–90 minutes, 16 sessions Topics: Financial education, RH information	Sample size: 615 intervention, 284 control/comparison Effects: *Increased mobility* *Increased autonomy when job searching* *Increased social support* *Increased number of savings accounts* *(formal and informal)* Decreased experience of sexual violence
Tap and Reposition Youth,^40^ 2001–2004	Kenya, urban	Quasi-experimental Aims: Reduce adolescents’ vulnerabilities to adverse social and RH outcomes by improving their livelihood options	Girls: 16–22 years old, out of school, married and unmarried Mentors: Must have worked in a profession related to counseling, social work, business, health care, community development, or business	15–25 girls, met weekly, 1–2 hours, 36 months’ duration Topics: Loan policies and procedures, business advice, gender issues, team building, adolescent RH, life skills, HIV/AIDS	Sample size: 222 intervention, 222 control/comparison Effects: *Increased earnings* *Increased number of savings accounts* *(formal and informal)* *Increased household assets* *Increased self-efficacy regarding condom* *use* Increased HIV knowledge Increased condom use Increased savings amount Increased RH knowledge Increased STI knowledge Improved attitudes toward girls' economic empowerment Improved attitudes toward GBV
iCuídate! Promueve tu Salud (Take Care of Yourself! Promote Your Health),^41^ 2002–2004	Mexico, urban	Randomized control trial Aims: Increase use of condoms and other contraceptives, decrease risky sexual behaviors of Mexican youth	Girls: 13–17 years old, in school Mentors: Trained	6–8 girls, met weekly, 6 hours, 2 consecutive Saturdays Topics: HIV/AIDS, health promotion, exercise, nutrition, substance abuse Also included: Boys/young men engagement	Sample size: 394 intervention,^d^ 314control/comparison^d^ Effects: *Increased condom use* *Increased contraceptive use* *Delayed sexual debut*
Choices,^42^^,^^43^ 2010	Nepal, rural	Quasi-experimental Aims: Improve gender equity among very young adolescents	Girls: 10–14 years old, in school, unmarried Mentors: 18–24 years old, graduate of the clubs, community members	Met weekly, 2 hours, 3 months’ duration Topic: Gender norms Also included: Boys/young men engagement	Sample size: 309 intervention (148 female, 161 male), 294 control/comparison (135 female, 159 male) Effects: *Improved attitudes toward GBV* *Improved beliefs regarding girls' education* *Changed perception of gender roles and* *norms*
Networks of Hope,^44^ 2012–2014	South Africa, rural	Randomized control trial Aims: For psychological intervention, mitigate mental health problems; for behavioral intervention, build participants' HIV knowledge and related skills; Both interventions were situated within broader OVC program offering educational and economic support to adolescents and their families	Girls: 14–17 years old; enrolled in OVC programming Mentors: Trained lay adult (for psychological intervention), trained young adult from community (for behavioral intervention)	18 girls, met weekly, 60–90 minutes, 13–16 weeks’ duration Topics: Life skills training, group therapy Also included: Boys/young men engagement, access and quality improvements of health services	Sample size: 785 intervention (375 female, 410 male), 229 control/comparison (110 female, 119 male) Effects: *Increased condom use* Decreased number of sex partners Delayed sexual debut
Siyakha Nentsha,^45^ 2008–2012	South Africa, rural	Quasi-experimental Aims: Powered to detect increased number of participants who save money and knowledge of government social grants, decrease social exclusion, increase interaction with formal financial institutions, improve HIV- prevention behaviors	Girls: Grade 10-11, in school Mentors: 20–24 years old, recent secondary school graduates, local	Met 2–3 days/week, 1 hour, 2 years’ duration Topics: Life skills training, nutrition, rights, financial literacy, job readiness Also included: Boys/young men engagement	Sample size: 359 female^e^, 356 male^e^ Effects: *Increased social support* *Increased number of savings accounts* *(formal and informal)* Decreased number of sex partners
Stepping Stones,^46^^,^^47^ 2003–2006	South Africa, rural	Randomized control trial Aims: Reduce incidence of HIV and HSV-2 and improve sexual practices among youth in South Africa's rural Eastern Cape Province	Girls: 16–26 years old, in and out of school Mentors: Same age or slightly older than girls, had post-school qualification, open-minded and gender sensitive	3 hours, 6–8 weeks’ duration Topics: Life skills training,GBV, HIV counseling/ treatment, comprehensive sex education Also included: Boys/young men engagement	Sample size: 1,409 intervention (715 female, 694 male), 1,367 control/comparison (701 female, 666 male) Effects: *Decreased HSV-2 incidence*Decreased HIV incidence Decreased pregnancies Decreased experience of physical violence Decreased negative mental health outcomes Decreased experience of sexual violence Decreased transactional sex Increased condom use Decreased number of sex partners Reduced drugs or alcohol misuse
Adolescent Development Program,^48^ 2009–2011	Tanzania, urban and rural	Randomized control trial Aims: Improve human capital and financial market participation of young women by providing vocational training and information on sex, reproduction, and marriage	Girls: 14–20 years old, in and out of school, married and unmarried Mentors: Adolescent leader from same community, few years older than girls	Met 5 days/week, 2 hours Topics: Sexual and RH, Life skills training, ivelihood training, microfinance/ microcredit, laws and rights	Sample size:3,179^f^ Effects: *Increased number of savings accounts* *(formal and informal)* *Changed perception of gender roles and* *norms* Decreased pregnancies Reduced STI symptoms Decreased experience of sexual violence Increased condom use Delayed sexual debut Reduced child marriage Increased school retention Increased employment Increased earnings Increased savings amount Increased HIV knowledge Improved attitudes toward child marriage Improved attitudes toward family sizes
Mabinti Tushike Hatamu!,^49^ 2012–2015	Tanzania, urban and rural	Quasi-experimental Aims: Reduce adolescent girls’ vulnerability to HIV, pregnancy, and violence	Girls: 10–19 years old, out of school, married and unmarried Mentors: 19–23 years old, similar to participants, recruited by local government or advertisement	10–15 girls, met 1–2 days/week, 32 months’ duration Topics: Life skills, income-generating activities, GBV education, education	Sample size: 291 intervention, 357 control/comparison Effects: *Increased condom use* *Increased health service utilization* *Increased utilization of violence treatment*, *support, and/or prevention services* *Increased vocational training* *Increased self-efficacy to assert opinions* *and concerns* *Increased social support* *Increased employment* *Increased RH knowledge* Decreased negative mental health outcomes Increased contraceptive use Decreased number of sex partners Delayed sexual debut Increased mobility Improved self-esteem Increased financial literacy
Young Citizens Program,^50^ 2004–2005	Tanzania, urban	Randomized control trial Aims: Increase youth participants' competencies so that they can plan and implement integrated health promotion activities that educate their communities and encourage them to take action toward HIV/AIDS prevention, testing, and treatment	Girls: 9–14 years old, in and out of school Mentors: Young adults, completed secondary school, previous experience in youth-related HIV activities	Met weekly, 2–3 hours, 28 weeks’ duration Topics: Social ecology, citizenship, community health, HIV/AIDS knowledge Also included: Boys/young men engagement	Sample size: 313 intervention,^g^ 300 control/ comparison^g^ Effects: *Increased self-efficacy to assert opinions* *and concerns*
Empowerment and Livelihoods for Adolescents: Uganda,^51^ 2008–2010	Uganda, urban and rural	Randomized control trial Aims: Bolster girls’ cognitive and noncognitive skills with: vocational skills training to enable adolescent girls to start small-scale income generating activities, life skills to build knowledge and reduce risky behaviors	Girls: 14–20 years old, in and out of school, married and unmarried Mentors: From community, slightly older than target girl population	Met 5 days/week, 2 years’ duration Topics: Life skills training, sexual and RH, vocational training, financial literacy	Sample size: 3,964 intervention, 2,002 control/comparison Effects: *Decreased experience of sexual violence* *Decreased pregnancies* *Increased condom use* *Reduced child marriage* *Increased employment* *Increased monthly expenditures* *Increased HIV knowledge* *Increased RH knowledge* *Improved attitudes toward child marriage* *Improved attitudes toward early pregnancy* *Improved attitudes toward family sizes* *Changed perception of gender roles and norms* Reduced STI symptoms Increased contraceptive use Increased health service utilization Increased school enrollment Increased earnings
Safe and Smart Savings,^39^^,^^52^ 2009–2011	Uganda, urban	Quasi-experimental Aims: Develop, pilot test, and roll-out individual savings accounts to girls belonging to girls'groups Program evaluation aims: Understand the social, economic, and health effects of participating in program activities	Girls: 10–19 years old, in and out of school, unmarried Mentors: 20–35 years old, reside in same community as girls in group, interest in working with vulnerable adolescent girls	15–25 girls, met weekly, 30–90 minutes, 16 sessions Topics: Financial education, RH information	Sample size: 750 intervention, 312 control/comparison Effects: *Increased number of savings accounts* *(formal and informal)* *Increased HIV knowledge* *Improved attitudes toward GBV* Increased HIV testing Increased mobility Increased autonomy when job searching Increased social support Decreased experience of sexual violence
Suubi Project,^53^^–^^58^ 2005–2016	Uganda, rural	Randomized control trial Aims: Suubi: Improve health, mental health, and life chances of AIDS-orphaned adolescents through microfinance and economic empowerment Suubi-Maka: Improve orphaned adolescents' attitudes toward HIV- preventive practices and future cash savings over time, as well as increase their cash savings Suubi+Bridges: Develop ability to identify future goals and educational aspirations by building their self- esteem; Improve school attendance and grades, encourage hopefulness, enhance safe sex decision making, and decrease sexual risk-taking behavior	Girls: 11–17 years old, in school, unmarried Mentors: University students, tried to recruit graduates of program	7 girls maximum Suubi: Monthly, 1–2 hours, 12 sessions Suubi-Maka: Not specified Suubi+Bridges: Monthly, 1 hour, 9 months’ duration Topics: Child savings accounts, financial literacy, asset building, life skills, HIV prevention Also included: Boys/young men engagement	Sample size: Suubi: 135 intervention (82 female, 53 male), 142 control/comparison (75 female, 67 male) Suubi-Maka:179 intervention (117 female, 62 male), 167 control/comparison (108 female, 59 male) Suubi+Bridges: 913 intervention (398 female, 515 male), 497 control/comparison (228 female, 269 male) Effects: *Improved self-rated health* *Improved self-esteem* *Increased savings amount* *Increased HIV knowledge* *Affected their perceived* *vulnerability to HIV/AIDS* *Increased concerns about unprotected sex* *Improved attitudes toward girls' economic* *empowerment*
Exploring the World of Adolescents,^59^ 2006	Vietnam, urban and rural	Randomized control trial Aims: Increase knowledge about HIV, STIs, and pregnancy and contraceptives; improve perceptions related to condom use and abstinence; increase condom use response efficacy; decrease intention to engage in sex in the next 3 months	Girls: 15–20 years old, in and out of school, unmarried Mentors: Trained, from the community	10 girls, met weekly for 2 hours, 10 sessions Topic: Life skills training Also included: Boys/young men engagement, access and quality improvements of health services	Sample size: 281 intervention (149 female,132 male), 317 control/comparison (167 female, 150 male) Effects: Increased HIV knowledge Increased RH knowledge Increased STI knowledge
Focus on Kids,^59^^,^^60^ 2001–2003	Vietnam, urban and rural	Randomized control trial Aims: Increase knowledge about HIV, STIs, and pregnancy and contraceptives; improve perceptions related to condom use and abstinence; increase condom use response efficacy; decrease intention to engage in sex in the next 3 months	Girls: 15–20 years old, in and out of school, unmarried Mentors: Trained, from the community	10 girls, met weekly, 2 hours, 10 sessions Topic: Life skills training Also included: Boys/young men engagement, access and quality improvements of health services	Sample size: 317 intervention (167 female, 150 male), 281 control/comparison (149 female, 132 male) Effects: *Increased HIV knowledge* Increased RH knowledge Increased STI knowledge
Adolescent Girls' Empowerment Program,^61^^,^^62^ 2013–2016	Zambia, urban and rural	Randomized control trial Aims: Empower adolescent girls by instilling them with social, health, and economic assets that they can draw upon to reduce vulnerabilities and expand opportunities, thereby increasing their likelihood of completing school and delaying sexual debut and reducing the risks of early marriage, unintended pregnancy, and HIV acquisition	Girls: 10–19 years old, in and out of school, unmarried Mentors: 20–40 years old, completed grade 12, can speak and write in English, experienced	20–30 girls, met weekly, 1–2 hours, 3 years’ duration Topics: Life skills training, savings account, health vouchers Also included: Access and quality improvements of health services	Sample size: 3,104 intervention (1,043 safe space arm, 1,031 safe space+health voucher arm, 1,030 safe space+health voucher+savings account arm), 1530 control/comparison Effects: *Decreased transactional sex* *Increased condom use* *Delayed sexual debut* *Increased STI knowledge* *Improved attitudes toward GBV* Decreased HIV incidence Decreased HSV-2 incidence Increased mobility Increased number of savings accounts (formal and informal) Increased financial literacy
Regai Dzive Shiri Project,^63^^,^^64^ 2003–2007	Zimbabwe, rural	Randomized control trial Aims: Reduce incidence of HIV and HSV-2 and rates of unintended pregnancy, improve knowledge, attitudes, and behaviors related to gender issues, HIV, and sexual risk	Girls: 18–22 years old, in and out of school, married and unmarried Mentors: School leaver in the year between leaving school and starting university	20–30 girls, 4 weeks’ duration Topics: HIV prevention, self-awareness and communication, rural development (risk and body mapping, drama, storytelling, and role play) Also included: Boys/young men engagement, access and quality improvements of health services	Sample size: 2,319 intervention (1,241 female, 1,078 male),1,353 control/comparison (1,352 female, 1,001 male) Effects: *Decreased pregnancies* *Increased self-efficacy regarding condom* *use* *Increased self-efficacy to seek out HIV* *testing* *Increased RH knowledge* *Increased STI knowledge* *Increased concerns about unprotected sex* Decreased HIV incidence Decreased HSV-2 incidence Reduced STI symptoms Increased condom use Increased contraceptive use Increased health service utilization Decreased number of sex partners Increased awareness of marital-related rights
Shaping the Health of Adolescents in Zimbabwe (SHAZ!) Project,^65^ 2006–2008	Zimbabwe, urban	Randomized control trial Aims: Improve sexual and structural risk factors and decrease unintended pregnancy and HIV and HSV-2 incidence among adolescent female orphans	Girls: 16–19 years old, out of school, married and unmarried Mentors: Self-selected adults	25 girls, 4–6 weeks’ duration, additional 6 months duration for livelihoods component Topics: Life skills training, livelihoods, microgrants Also included: Access and quality improvements of health services	Sample size: 158 intervention, 157 control/comparison Effects: *Increased employment* *Decreased food insecurity* Decreased HIV incidence Decreased HSV-2 incidence Decreased pregnancies Decreased experience of physical violence Decreased experience of sexual violence Increased condom use Increased contraceptive use Decreased transactional sex Decreased number of sex partners Increased social support

Abbreviations: FP, family planning; GBV, gender-based violence; HSV-2, herpes simplex virus 2; RH, reproductive health; STIs, sexually transmitted infections.

a Italicized effects signify statistical significance [α=0.05]).

b Evaluation used cross-sectional surveys to collect baseline and end line data; although, the methodology report didn’t contain details on matching or follow-up. Based on the assumption that baseline and end line samples covered different people, we aggregated the number of respondents across both in the calculation.

c Though female genital mutilation/cutting (FGM/C) significantly increased for participants in control group compared to intervention arm, the study cites differing FGM traditions may be the reason, e.g., ages villages traditionally perform FGM/C. Difference between baseline and end line prevalence show most girls (>50%) in program villages entered program already circumcised, while most girls in control villages (<40%) were not. This suggests control villages perform FGM/C at later ages than program villages and the statistically significant difference-in-difference calculation between program and control villages might not be attributable to intervention.

d Evaluation reports more female than male participants (405 versus 303) but doesn’t report numbers of females/males in each arm.

e Evaluation provides sex-stratified demographic information/analyses; doesn’t report numbers of females/males in each arm.

f Total sample size for both intervention and control/comparison arm; evaluation doesn’t specify numbers for each.

g Evaluation controls for sex in multivariable models but doesn’t report numbers of females/males in each arm.

### Implementation Science Findings

Program design and the quality of implementation influences program effects. The replication of a program with proven efficacy may fail to have the same real-world effect if not implemented with fidelity to the original design. Despite this, research on design features is largely missing from the literature.^10^ We sought to fill this gap by collecting information on selected design features of programs in our sample. To note, not every publication provided the same amount of program design, planning, and implementation details. In addition, information was insufficient to compare the attributes of individual programs in our sample and rigorously assess success factors.

**FIGURE 2.**
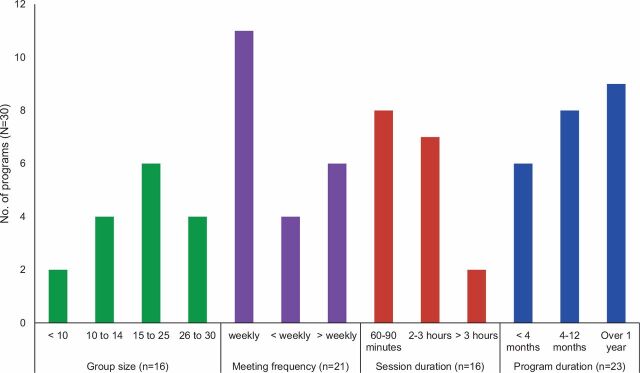
Frequency of Selected Design Features of Community-Based Girl Group Programs

The amount of information on design features varied considerably. Of 30 programs, 16 reported on the size and 21 on the frequency of group meetings (Figure 2). The most common group size was 15 to 25 girls, who typically met in groups weekly for 1 to 3 hours. Although no clear pattern emerged on program lifespan, nearly half of those reporting this information operated for more than a year. Information on girls’ actual participation is needed to assess exposure; however, less than half the programs reported this. According to that information, programs retained an average of 75% of participants (definitions of retention varied from 50% to 100% of sessions).

**FIGURE 3.**
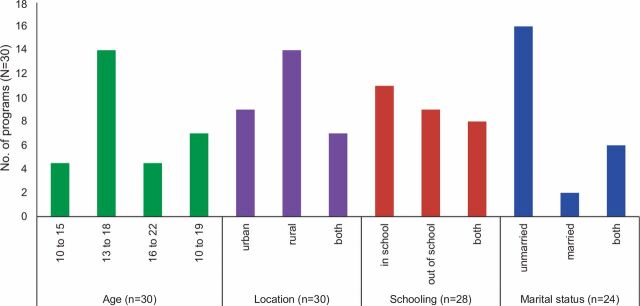
Frequency of Participants’ Features in Community-Based Girl Group Programs

The information provided about coverage revealed that the largest number of programs targeted unmarried girls aged 13–18 years who were both in school and not in school; more programs occurred in rural than in urban areas (14 rural, 9 urban, 7 in both; Figure 3). The limited details about which girls the programs tried to reach made it difficult to determine if they targeted girls at highest risk of the outcomes they sought to address. For example, for HIV prevention, were the girls who learned about condom self-efficacy the same girls having unprotected sex with an older partner? For child marriage prevention, were the girls who learned about the risks of early marriage the girls most likely to be married off?

Around one-third of programs reported that they adapted aspects of program design to different girl segments. Underscoring the importance of recognizing adolescent girls’ heterogeneity, participation and program effects varied between types of girl. The subset of evaluations that disaggregated participation rates by girl segment (e.g., by age [10–14 years, 15–19 years], schooling and marital status) found that younger girls attended more frequently than older girls and unmarried girls attended more frequently than married girls, whose responsibilities and social expectations differ. The variation in participation points to the importance of disaggregating design features and evaluation results for programs that target large, diverse groups of girls—for instance, girls aged 10 to 19 years, or both girls in school and not in school—which characterized around half the programs in the sample.

**FIGURE 4.**
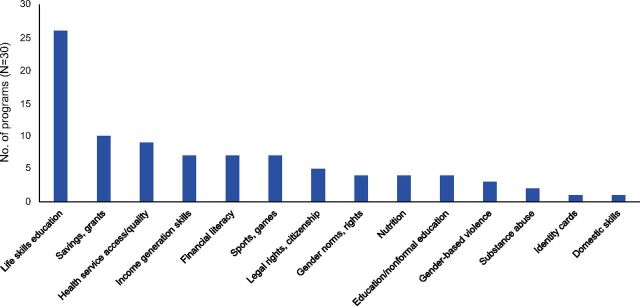
Types of Girl-Centered Content in Safe-Space Style Programs^a^ ^a^Nearly all programs addressed multiple content areas.

CBGG programs used a variety of interventions to deliver content to girls (Figure 4). In addition to serving as a base for referrals and community engagement, enhancements may have influenced outcomes for girls. All but 4 programs included content on life skills. Only 2 of the 30 programs restricted themselves to a single content area; in 17 programs, mentors combined life skills training with activities related to economic and financial outcomes, like income generation skills, financial literacy training, and access to microsavings or cash transfers. Nearly one-third of programs included activities to strengthen access to and/or quality of health services, such as health vouchers. Programs also included recreational activities such as sports and games. Across different content areas, regular group meetings built social support with mentors and peers to reduce social isolation. To complement the girl-centered content and promote an enabling environment, program staff used varied tactics to engage community members, local leaders, families, and male partners.

Programs recruited female mentors who often were local to the program community. Although most mentors were lay people, 4 programs recruited professionals from relevant fields, such as teachers and program staff. A schooling qualification was common, primarily secondary school graduation or the local equivalent. The mentors received specific training for their role; among those reporting this information, mentor training lasted 5 days or longer, and a few programs conducted refresher training following the initial mentor training. Despite the central role of mentors in this program model, reports rarely included details like selection criteria, job descriptions, and training strategies.

### Program Effects

#### Distribution of Program Effects by Outcome Domain

**FIGURE 5.**
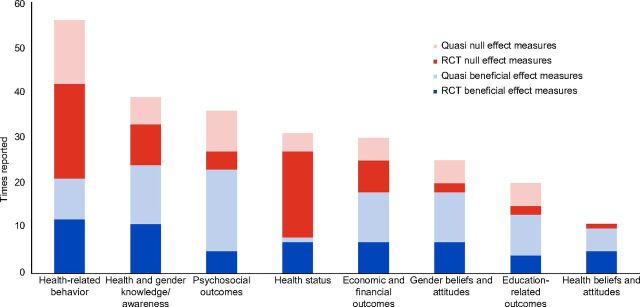
Distribution of Community-Based Girl Group Program Effects by Outcome Domain^a^^,^^b^ Abbreviations: Quasi, quasi-experimental; RCT, randomized controlled trial.^a^Beneficial = Reported statistically significant (α=0.05).^b^Null = Reported non-significant (α=0.05).

**Assessment of Evidence Base**. Table 1 presents the total number of times that evaluations measured the effects in each outcome domain across programs. Figure 5 shows the amount of evidence available for each domain and the number of times those outcomes were measured; a program contributes 1 “time reported” (i.e., the y-axis) per effect (e.g., increased mobility). Evaluations measured multiple outcomes and, therefore, could be counted more than once per domain. For example, an evaluation could contribute 2 times reported to the psychosocial outcomes domain if its evaluation measured both mobility and social support. Health-related behavior was the most frequently measured domain, followed by knowledge and awareness on health and gender, then psychosocial outcomes and health status.

Figure 5 also shows the reported beneficial and null effect measures. In absolute terms, evaluations reported the largest number of beneficial measures for knowledge and awareness on health and gender, followed by psychosocial outcomes, then health-related behavior, economic and financial outcomes, gender beliefs and attitudes, education-related outcomes, health beliefs and attitudes, and health status.

The number of beneficial effect measures in each outcome domain is not strictly comparable because the quantity of reported measures varied between domains. For example, programs had more opportunity to display changes in health-related behaviors than education-related outcomes because more reported on the former than the latter. To avoid biased interpretation, it is more informative to compare the number of beneficial measures with the overall number of measures (beneficial+null) within each domain. In relative terms, programs reported more (i.e., >50%) beneficial measures than null ones for beliefs and attitudes about health and gender, education-related outcomes, psychosocial outcomes, knowledge and awareness on health and gender, and economic and financial outcomes. Programs reported fewer (<50%) beneficial measures for health-related behaviors and health status. Results for each domain are detailed below in order of the proportion of beneficial effect measures, from most to least relative benefits.

**Review of Evaluations and Their Effects**. Figure 5 differentiates effects by study design. It indicates the likelihood that results are generalizable given that results of RCTs are more robust than other designs, although all impact evaluations in the sample met our criteria for rigor (as described above). To note, most effects on health status, health-related behavior, and knowledge and awareness were measured in RCTs, and quasi-experimental studies focused heavily on psychosocial outcomes. Across all outcome domains, quasi-experimental studies reported more beneficial measures than RCTs.

### Program Effects by Outcome Domain

#### Health Attitudes and Beliefs

Programs focused on topics that threaten girls’ growth and development, such as early pregnancy and female genital mutilation/cutting, to shift their attitudes about their health. Seven programs sought to change girls’ health beliefs and attitudes; in total, 91% of the effect measures reported a significant change in the intended direction, making this the domain with the highest proportion of beneficial measures (Table 1).

The evaluation of *Ishraq*, a program in Upper Egypt to empower adolescent girls and improve their knowledge and attitudes to promote healthy and safe transitions to adulthood, reported that it improved girls’ attitudes toward performing female genital mutilation/cutting on their daughters in the future.^22^ *Regai Dzive Shiri* was a cluster RCT to reduce HIV among Zimbabwean youth who were in school and not in school through work with community members, clinic staff, and young people. Its evaluation reported that it increased girls’ concerns about unprotected sex (Table 2).^63^^,^^64^

#### Gender Attitudes and Beliefs

Programs aimed to shift participants’ beliefs and attitudes toward a more egalitarian stance by addressing practices like child marriage and gender-based violence (GBV). Twelve programs aspired to change girls’ attitudes and beliefs regarding gender; collectively, 72% of this domain’s effect measures were beneficial (Table 1).

Program evaluations reported improvements in girls’ attitudes or perceptions toward GBV, child marriage, and gender roles and norms. For example, an evaluation of Choices, a curriculum-based program to shift gender-related attitudes and behaviors in rural Nepal, reported that the program reduced girls’ acceptance of GBV.^42^ An evaluation of Better Life Options, a life skills education program in Uttar Pradesh, India, reported that it improved girls’ attitudes toward child marriage (Table 2).^30^

#### Education-Related Outcomes

Programs aimed to improve education-related behaviors (e.g., school enrollment) and skills (e.g., numeracy). Evaluations of 10 programs assessed education-related effects and reported beneficial effects 65% of the time they were measured (Table 1).

Overall, program evaluations reported improvements in girls’ numeracy skills and increases in school enrollment. In Ethiopia, *Biruh Tesfa* worked with marginalized girls to improve education-related outcomes. Among participants with no formal schooling, the evaluation reported that the program increased girls’ numeracy and literacy scores.^27^ An evaluation of the scale-up of *Ishraq* reported that girls’ reading comprehension and multiplication skills improved (Table 2).^23^

#### Psychosocial Outcomes

Evaluations used a variety of indicators to track psychosocial outcomes, which include self-efficacy, mobility, autonomy, and social support, as well as experience of gender discrimination. Evaluations of 19 programs reported psychosocial outcomes, and 64% of these effect measures were statistically significant. Proportionally, more than half of the measures of girls’ self-efficacy regarding SRH behaviors, such as condom use and HIV testing, social support, and assertiveness were beneficial (Table 1).

The evaluation of BRAC’s Employment and Livelihood for Adolescents program—which aimed to reduce child marriage, keep girls in school, and increase girls’ peer socialization in Bangladesh through income generation and group activities—reported that it increased girls’ mobility.^19^ The Young Citizens Program in Tanzania used education and community mobilization to strengthen very young adolescents’ agency in planning and implementing health promotion activities related to HIV. The evaluation reported that it increased girls’ efficacy to assert their thoughts and opinions with peers and adults.^50^ Also in Tanzania, the evaluation of *Mabinti Tushike Hatamu!*, a program to reduce the vulnerability of girls who were not in school, reported that it increased the number of girls who said that community leader requested their opinion (Table 2).^49^

#### Knowledge and Awareness about Health and Gender

Seventeen programs aspired to improve girls’ knowledge about health topics, like HIV and marriage-related rights. Their evaluations reported beneficial effect measures 62% of the time, with more success on knowledge measures related to health (63%) than to gender (50%). Evaluations reported more beneficial effects regarding HIV and reproductive health knowledge than regarding sexually transmitted infection (STI) and menstrual regulation knowledge and awareness of marriage-related rights (Table 1).

An evaluation of the *Suubi* & Bridges Project, a Ugandan peer mentorship program to protect AIDS-orphaned adolescents against HIV and STIs by providing culturally appropriate HIV information, reported that the program increased HIV knowledge.^53^ In India, Promoting Change in Reproductive Behavior (known as PRACHAR) in Bihar aimed to increase contraceptive use and delay pregnancy. Although it reportedly increased reproductive health knowledge, it did not succeed in delaying first pregnancy (Table 2).^32^

#### Economic and Financial Outcomes

Evaluations of 15 programs measured economic and financial outcomes and reported beneficial measures 60% of the time. The effects with the highest proportion of beneficial measures were increasing girls’ employment, savings accounts, and household assets, as well as decreasing food insecurity. The results related to girls’ earnings were mixed (i.e., 50% beneficial), and according to the evaluations, no program reduced dowry practices (Table 1).

The evaluation of the Shaping the Health of Adolescents in Zimbabwe (known as SHAZ!) Project, which aimed to prevent HIV among adolescent girls through structural interventions, reported that it increased girls’ receipt of their own income.^65^ *Siyakha Nentsha* was a 2-armed intervention in South Africa to improve girls’ and boys’ economic well-being that provided training on life skills, HIV/STI prevention, and social capital building. One arm also received household financial management and small business planning (financial education arm) and another received training in sexuality, reproductive rights, and stress and violence reduction (stress management arm). The evaluation reported that *Siyakha Nentsha* increased the number of savings accounts (stress management arm) and girls’ interaction with banks (financial education arm) (Table 2).^45^

#### Health-Related Behavior

Nineteen programs sought to improve behaviors, especially those related to SRH (e.g., transactional sex, condom use). Collectively, 38% of the effect measures reported for this domain were beneficial. Effects that were beneficial every time they were measured included: increased secondary abstinence; menstrual hygiene management; and violence treatment, support, and/or prevention services. One-third of the programs included complementary activities to improve access to and quality of health services; however, evaluations reported that health service utilization significantly increased only 50% of the time it was measured. Child marriage significantly decreased nearly 40% of the times it was measured according to evaluation reports. Most program evaluations reported null effects for girls’ number of sex partners, transactional sex, condom use, sexual debut, and contraceptive use (Table 1).

Although well under half of this domain’s measures were beneficial (38%), individual programs reported notable changes in health behavior. The Bangladeshi Association for Life Skills, Income, and Knowledge for Adolescents (known as BALIKA) program aimed to reduce child marriage using weekly girl-only meetings combined with different topics across 3 study arms. The program’s evaluation reported that it decreased the odds of child marriage across all 3 arms: girls in the tutoring arm had the lowest odds of marriage before the age of 18.^18^ The evaluation of Networks of Hope, a multi-arm South African program to reduce HIV risk by improving psychological and behavioral outcomes, reported that it increased girls’ consistent condom use.^44^ In a rare example of a longitudinal effect measure, the evaluation of a Mexican program, *Cuídate*! *Promueve tu Salud*, reported that it increased participants’ age at first sex in a 4-year follow-up survey (Table 2).^41^

#### Health Status

Evaluations of 11 programs (8 were RCTs) assessed changes in health status using self-reports and biomarkers. Few evaluations reported statistically significant improvements in health status effects, such as experience of physical violence and HSV-2 incidence; of the times evaluations measured improved health status, only 26% were beneficial. The 4 programs that measured HIV incidence did not report a decrease. Evaluations reported that measures of decreasing girls’ experience of sexual and physical violence were null more often than beneficial. No programs reported mental health improvements or STI reductions (Table 1).

Stepping Stones is a program to improve sexual health with participatory learning to build knowledge, risk awareness, and communication skills. Its evaluation reported that the program reduced HSV-2 incidence.^46^ The evaluation of Growing Up Safe & Healthy in Bangladesh, which used a multipronged delivery model including male groups, female groups, and community mobilization, reported it decreased girls’ experience of physical and/or sexual violence (Table 2).^20^

## DISCUSSION

The expanding evidence base on CBGGs enables an analysis of their effects across programs and countries. Notably, the size of the evidence base varies for each outcome domain and limits comparability between the summaries of impact. The variation reflects funding patterns for CBGGs, which are dominated by HIV prevention, explaining the preponderance of health behavior measurement. The results only describe what was measured, which may or may not encompass all the changes resulting from the programs. For these reasons, the relative assessment, which indicates how the program did in relation to its aims, is more informative than the absolute assessment.

Different types of study designs in our sample yielded different types of results. In general, the RCTs emphasized outcomes that could be objectively measured in the domains of health status and behavior (albeit mostly self-reported). The quasi-experimental evaluations tended to emphasize outcomes that are more complex to measure, such as psychosocial outcomes and attitudes.

Evaluations of programs using CBGGs reported improvements in girls’ attitudes and beliefs about gender and health; boosts in educated-related outcomes, such as numeracy and school enrollment; and increases in girls’ economic and psychosocial assets. They also reported positive effects on knowledge and awareness about health and gender. In general, these results suggest that CBGGs appear to have more potential to impact individual outcomes than outcomes that rely on a group. Theoretically, all of these are along the causal pathway to good health.

Despite the reported boost that programs gave mediating factors that theoretically improve health behavior and health status, reports of program performance on behavior and health status is mixed. For instance, condom use increased less than half the times measured (5 of 11) and contraceptive use increased one-third of the times measured (3 of 9). Only one-quarter of reported measures of girls’ health status (e.g., experience of physical or sexual violence, fertility, STI incidence) were statistically significant, and child marriage practices improved just under half the time that evaluations measured them (3 of 8 times). These results are not unexpected given that attitudes and knowledge change faster than behavior and, ultimately, health status.^66^

The theoretical pathway to health behavior change is well-documented and offers possible reasons that changes in mediating factors did not consistently translate into behavior change and better health within evaluation time frames. Explanations relate to girls’ locus of control and program and study designs.^67^^,^^68^ First, the main benefits of CBGG programs reflect changes that are internal to girls—for example, attitudes toward child marriage, demand for health services, self-esteem, and literacy. In general, effects are weaker on outcomes that rely on factors external to girls—such as condom use, HIV testing, child marriage, and health service utilization. This difference may reflect inequitable interpersonal relationships; weak access to transport, finances, services; and other socioeconomic factors that impede girls’ ability to exercise their voice, choice, and control over behaviors and, consequently, their health and well-being. Notably, most programs with CBGGs included activities to engage community members that theoretically have the potential to reduce barriers to behavior change. However, details on community engagement and its influence on girl-level outcomes was rarely reported in the impact evaluations in our sample.

The main CBGG program benefits to girls appear to be internal changes, such as attitudes toward child marriage and self-esteem.

Second, related to study and program design, participation rates varied between different subpopulations of girls. This may have led to mixed effects for different girl segments that reported results may have masked. For instance, if younger girls participate more in meetings than older girls, they may derive more benefits that may not appear in a summary effect measure.^61^ Zambia’s Adolescent Girls’ Empowerment Program documented more participation among younger and rural participants than older and urban ones; not surprisingly, the evaluation found that younger unmarried girls benefited more than older married girls. ^61^ Given their central role in delivering content in CBGGs, mentor performance is another important mediator of effects masked by aggregated results. The scant evidence available on mentor quality indicates that mentors’ own characteristics and the quality of their performance is a major source of variability in girls’ participation and impact. ^61^ Aggregated results of impact evaluations of programs for diverse groups of girls (e.g., girls aged 10–19 years in school and not in school) and mentors risk eclipsing effects for some subsets of participants in the absence of disaggregation.

Third, related to study design, when and what outcomes the impact evaluations measured influenced our results. The types of outcome measures that dominated impact evaluations and the data collection instruments used may not have been adequate to capture the types of changes that CBGGs are most likely to bring about. In addition, most evaluations captured short-term effects after programs ended; they rarely returned to measure long-term impact. A few notable exceptions include Mexico’s *Cuídate*! *Promueve tu Salud*, where researchers returned 4 years after activities ended to assess the durability of effects. Most young adolescents are not yet sexually active; given the possibility that younger participants attend more regularly than older ones, it is conceivable that the most active CBGG participants faced the least behavioral risk within evaluation time frames. This would limit the likelihood of evaluations finding sexual behavioral and health effects. Long-term follow-up would reveal if benefits endure and these girls reduce behavioral and health risks as they age or if benefits wash out over time.

### Limitations

The summary of CBGGs effects is informative. However, limited evidence and the lack of comparability between studies make these results preliminary. The small size of the evidence base, as well as the tremendous variability in the study designs, implementation features, and outcomes measured, prevented us from conducting a meta-analysis, which would have enabled us to assess effects across programs. More evidence, including from implementation science research, would shed light on the most promising design features, making the practical implications of impact evaluation results clearer. In addition, too few multicomponent studies compared different combinations of interventions and content to enable a detailed assessment of attribution. For example, we could not assess the effect of group-level changes resulting from community engagement activities that may have influenced girl-level effects.

Although the literature review was comprehensive, it was not a systematic review; as a result, we may have missed relevant evidence. The tendency to favor positive results in publications may have led us to overestimate the benefits of CBGGs. Additionally, evaluations relied heavily on self-reported information, which introduces the possibility of social-desirability and recall biases. Finally, although the RCTs were designed to reduce the risk of selection bias, it is possible that girls who joined CBGG programs and participated regularly differed from nonparticipants and dropouts in ways that influenced the likelihood of impact.

## SUMMARY AND IMPLICATIONS FOR PROGRAMS, POLICIES, AND RESEARCH

Most CBGGs in our sample included 20 (± 5) girls, met weekly for more than an hour, and lasted for a year or longer; they frequently combined life skills training with content to promote economic and financial outcomes, such as financial literacy or access to microfunds/bank accounts. Providing girls with an opportunity to build social connections with peers and mentors in a safe space has intrinsic value. Furthermore, the evaluations in this review indicate that programs with these characteristics can use locally recruited female mentors to build girls’ economic and psychosocial assets; improve their attitudes, beliefs, knowledge, and awareness on health and gender; and enhance education-related outcomes. Enhancements found in many programs like community engagement and health services strengthening may have influenced the impact of the CBGGs on girls.

These results suggest that CBGGs have more potential for benefits that may contribute to girls’ empowerment than to their health in the near term. Girls’ empowerment, which encompasses their voice, choice, and control over key aspects of their lives, can increase their likelihood of growing into successful, healthy adults.^69^^,^^70^ Empowerment is a critical development goal in itself that can position girls to make decisions and affect outcomes of importance to themselves, their families, and their communities—especially when the social environment supports these changes. Beyond direct benefits, a girl’s empowerment can affect other aspects of her health and well-being. As girls gain voice, choice, and control, in the context of an enabling environment, over time they may benefit from improved outcomes, including delayed marriage and pregnancy, reduced violence, better health, more education, and greater learning. Ultimately, these positive shifts may improve girls’ and women’s well-being and life chances and reduce the intergenerational transmission of poverty.

These results have implications for research. As the evidence on CBGGs grows, future studies should assess the types of girl-level changes CBGG programming is most likely to bring about, including neglected outcomes such as mental health and nutrition. More evidence would enable a rigorous comparison—such as a meta-analysis—of how this program model performs on key outcomes, like child marriage, relative to other interventions, which would make an important contribution to the evidence base. Ensuring impact evaluations are robust and illuminate program methodology and outcome measurement is paramount; using comprehensive research reporting standards and guidelines can help.^71^^,^^72^ Future evaluations also should consider using triangulation techniques (i.e., comparing self-reported information to records) or supplemental data collection methods (e.g., direct observation) to validate self-reported responses.

Questions remain about how to use the platform that CBGGs provide to best protect and empower adolescent girls in their communities. How do effects vary between different girl segments, and which girl segments are the most important to target (e.g., unmarried, younger girls) for broad changes over time and into the next generation? This program delivery model has salience for married girls, who often are socially isolated and facing high risk, but few impact evaluations included them. Other questions on the effects of CBGGs include how durable effects are and if they wash out over time.

Questions remain about how to use CBGG platforms to best protect and empower adolescent girls in their communities.

Given increased investment in CBGGs, evidence is needed on their scalability, such as the minimum package of elements required to have an effect. Evaluations of layered combinations of interventions would be informative. Other questions on designing for scale relate to the optimal design model in real-world conditions: the ideal dosage or level of exposure; duration; group size and composition; mentor qualifications and skills; and the cost of retaining quality, effectiveness, and cost-effectiveness as coverage expands. For an enabling environment, how can girl programs effectively engage and mobilize boys, men, and other community members? What are effective tactics for institutionalizing CBGGs within existing government systems, including health systems, for sustainability?

Community-based programming can offer a way to reach adolescents who are out of school, disengaged from formal labor markets, and who rarely use health services. Given that excluded adolescents often face the highest risks of the worst outcomes, assessing the potential of targeted CBGG programs to reach these subpopulations is vital to understand their potential for equity and cost-effectiveness. More impact evaluations should disaggregate results to reflect adolescent heterogeneity, as well as determining what add-ons are required to reach and retain the most excluded girls.

## Notes

**Peer Reviewed**

## References

[1] The World Factbook 2020: Country Comparison: Median Age. Central Intelligence Agency website. Accessed May 19, 2020. https://www.cia.gov/library/publications/resources/the-world-factbook/fields/343rank.html

[2] United Nations. *The Sustainable Development Goals Report 2018*. New York: United Nations; 2018. Accessed May 19, 2020. https://unstats.un.org/sdgs/files/report/2018/TheSustainableDevelopmentGoalsReport2018-EN.pdf

[3] World Health Organization (WHO). *Health in 2015: From MDGs Millennium Development Goals to SDGs Sustainable Development Goals*. Geneva: WHO; 2015. Accessed June 8, 2020. https://apps.who.int/iris/bitstream/handle/10665/200009/9789241565110_eng.pdf

[4] Child marriage around the world. Girls Not Brides website. Accessed May 19, 2020. https://www.girlsnotbrides.org/where-does-it-happen/

[5] United Nations Education, Scientific and Cultural Organization (UNESCO) Institute for Statistics (UIS). *One in Five Children, Adolescents and Youth is Out of School*. UIS Fact Sheet 48. Montreal: UIS; 2018. Accessed May 19, 2020. http://uis.unesco.org/sites/default/files/documents/fs48-one-five-children-adolescents-youth-out-school-2018-en.pdf

[6] World Health Organization (WHO). *Making Health Services Adolescent Friendly: Developing National Quality Standards for Adolescent Friendly Health Services*. Geneva: WHO; 2012. Accessed May 19, 2020. https://www.who.int/maternal_child_adolescent/documents/adolescent_friendly_services/en/

[7] Saul J, Bachman G, Allen S, Toiv NF, Cooney C, Beamon TA. The DREAMS core package of interventions: a comprehensive approach to preventing HIV among adolescent girls and young women. PLoS One. 2018;13(12):e0208167. 10.1371/journal.pone.0208167. 30532210 PMC6285267

[8] Temin M, Amin S, Ngo TD, Psaki S. How to give adolescent girls voice, choice, and control. Stanford Social Innovation Review. December 17, 2018. Accessed May 19, 2020. https://ssir.org/articles/entry/how_to_give_adolescent_girls_voice_choice_and_control#

[9] Plourde KF, Ippoliti NB, Nanda G, McCarraher DR. Mentoring interventions and the impact of protective assets on the reproductive health of adolescent girls and young women. J Adolesc Health. 2017;61(2):131–139. 10.1016/j.jadohealth.2017.03.002. 28528208

[10] Haberland NA, McCarthy KJ, Brady M. A systematic review of adolescent girl program implementation in low-and middle-income countries: evidence gaps and insights. J Adolesc Health. 2018;63(1):18–31. 10.1016/j.jadohealth.2017.11.294. 29434004

[11] Marcus R, Gupta-Archer N, D’Arcy M, Page E. *Gender & Adolescence: Global Evidence (GAGE) Rigorous Review: Girls’ Clubs, Life Skills Programmes and Girls’ Well-Being Outcomes*. London*:* GAGE; 2017. Accessed May 19, 2020. https://www.gage.odi.org/wp-content/uploads/2019/01/GAGE-Girls-Club-Report-FINAL.pdf

[12] Rankin K, Jarvis-Thiébault J, Pfeifer N, et al. Adolescent sexual and reproductive health: An evidence gap map. New Delhi, India: International Initiative for Impact Evaluation; Accessed June 8, 2020. https://www.3ieimpact.org/evidence-hub/publications/evidence-gap-maps/adolescent-sexual-and-reproductive-health-evidence-gap

[13] Kwauk C, Braga A, Kim H, Dupuy K, Bezu S, Knudsen A. *Non-Formal Girls’ Life Skills Programming: Implications for Policy and Practice*. Washington, DC: Center for Universal Education at The Brookings Institution; 2018. Accessed May 19, 2020. https://www.brookings.edu/wp-content/uploads/2018/06/Non-formal-girls-life-skills-programming_A4.pdf

[14] Cluver LD, Orkin MF, Yakubovich AR, Sherr L. Combination social protection for reducing HIV-risk behavior among adolescents in South Africa. J Acquir Immune Defic Syndr. 2016;72(1):96–104. 10.1097/qai.0000000000000938. 26825176 PMC4839503

[15] Toole, R. *Reducing Pregnancy Among Adolescents*. Cambridge, MA: Abdul Latif Jameel Poverty Action Lab; 2018. Accessed May 19, 2020. https://www.povertyactionlab.org/sites/default/files/publications/reducing-pregnancy-among-adolescents.pdf

[16] Alvarado G, Skinner M, Plaut D, Moss C, Kapungu C, Reavley N. *A Systematic Review of Positive Youth Development Programs in Low- and Middle-Income Countries*. Washington, DC: YouthPower Learning, Making Cents International; 2017. Accessed May 19, 2020. https://www.youthpower.org/sites/default/files/YouthPower/files/resources/SystematicReview%20FINAL%209-26-17%20compress.pdf 10.1016/j.jadohealth.2019.01.024 31010725

[17] International Initiative for Impact Evaluation. Adolescent Sexual and Reproductive Health Evidence Gap Map. Updated February 2, 2017. Accessed May 19, 2020. http://gapmaps.3ieimpact.org/evidence-maps/adolescent-sexual-and-reproductive-health-evidence-gap-map

[18] Amin S, Ahmed J, Saha J, Hossain I, Haque E. *Delaying Child Marriage Through Community-Based Skills-Development Programs For Girls*. *Results From a Randomized Controlled Study in Rural Bangladesh*. Dhaka, Bangladesh: Population Council; 2016. Accessed May 19, 2020. https://www.popcouncil.org/uploads/pdfs/2016PGY_BALIKA_EndlineReport.pdf

[19] Shahnaz R, Karim R. *Providing Microfinance and Social Space to Empower Adolescent Girls: An Evaluation of BRAC’s ELA Centres*. Dhaka, Bangladesh: BRAC Research & Evaluation Division; 2008. Accessed May 19, 2020. http://www.esocialsciences.org/Download/repecDownload.aspx?fname=Document11062010590.9487116.pdf&fcategory=Articles&AId=2549&fref=repec

[20] Naved RT, Amin S, eds. *Impact of SAFE Intervention on Sexual and Reproductive Health and Rights and Violence Against Women and Girls in Dhaka Slums*. Dhaka, Bangladesh: Population Council; 2014. Accessed May 19, 2020. https://www.popcouncil.org/uploads/pdfs/2014PGY_SAFE-Report.pdf

[21] Amin S, Suran L. *Program Efforts to Delay Marriage Through Improved Opportunities: Some Evidence from Rural Bangladesh*. Dhaka, Bangladesh: Population Council; 2005. Accessed May 19, 2020. https://paa2005.princeton.edu/papers/51141

[22] Brady M, Assaad R, Ibrahim BL, Salem A, Salem R, Zibani N. *Providing New Opportunities to Adolescent Girls in Socially Conservative Settings: The Ishraq Program in Rural Upper Egypt*. Cairo, Egypt: Population Council; 2006. Accessed May 19, 2020. http://www.cpcnetwork.org/wp-content/uploads/2014/04/IshraqFullReport.pdf

[23] Sieverding M, Elbadawy A. Empowering adolescent girls in socially conservative settings: impacts and lessons learned from the Ishraq program in rural upper Egypt. Stud Fam Plann. 2016;47(2):129–144. 10.1111/j.1728-4465.2016.00061.x. 27285424

[24] Ringler K. *A Review of the Ishraq Program’s Quasi-Experimental Impact Evaluation* [master’s thesis]. Minneapolis: University of Minnesota; 2009. Accessed May 19, 2020. https://conservancy.umn.edu/handle/11299/50227

[25] Selim M, Abdel-Tawab NG, Elsayed K, El Badawy A, El Kalaawy H. *The Ishraq Program for Out-of-School Girls: From Pilot to Scale-up*. Cairo, Egypt: Population Council; 2013. Accessed May 19, 2020. https://www.popcouncil.org/uploads/pdfs/2013PGY_IshraqFinalReport.pdf

[26] Erulkar AS, Muthengi E. Evaluation of Berhane Hewan: a program to delay child marriage in rural Ethiopia. Int Perspect Sex Reprod Health. 2009:6–14. 10.1363/ifpp.35.006.09. 19465343

[27] Medhin G, Erulkar A. Evaluation of a safe spaces program for girls in Ethiopia. Girlhood Stud. 2017;10(1):107–125. 10.3167/ghs.2017.100108

[28] Erulkar A, Ferede A, Girma W, Ambelu W. Evaluation of “Biruh Tesfa” (Bright Future) program for vulnerable girls in Ethiopia. Vulnerable Child Youth Stud. 2013;8(2):182–192. 10.1080/17450128.2012.736645

[29] Erulkar A, Medhin G. *Evaluation of Health and Education Impacts of a Girls’ Safe Spaces Program in Addis Ababa Ethiopia*. Addis Ababa, Ethiopia: Population Council; 2014. Accessed May 19, 2020. https://www.popcouncil.org/uploads/pdfs/2014PGY_HealthEducImpactsSafeSpaces.pdf

[30] Acharya R, Kalyanwala S, Jejeebhoy SJ, Nathani V. *Broadening Girls’ Horizons: Effects of a Life Skills Education Programme in Rural Uttar Pradesh*. New Delhi, India: Population Council; 2009. Accessed May 19, 2020. https://www.issuelab.org/resource/broadening-girls-horizons-effects-of-life-skills-education-programme-in-rural-uttar-pradesh.html

[31] Santhya K, Haberland N, Das A, et al. *Empowering Married Young Women and Improving their Sexual and Reproductive Health: Effects of the First-time Parents Project*. New Delhi, India: Population Council; 2008. Accessed May 19, 2020. https://www.ohchr.org/Documents/Issues/Women/WRGS/ForcedMarriage/NGO/PopulationCouncil23.pdf

[32] Pandey N, Jejeebhoy SJ, Archarya R, Singh SK, Srinivas M. *Effects of the PRACHAR Project’s Reproductive Health Training Programme For Adolescents: Findings From a Longitudinal Study*. New Delhi, India: Population Council; 2016. Accessed May 19, 2020. https://www.popcouncil.org/uploads/pdfs/2016PGY_PracharReport.pdf

[33] Daniel EE, Masilamani R, Rahman M. The effect of community-based reproductive health communication interventions on contraceptive use among young married couples in Bihar, India. Intl Fam Plan Perspect. 2008;34(4):189–197. 10.1363/ifpp.34.189.08. 19201679

[34] Wilder J, Masilamani R, Daniel E. *Promoting Change in the Reproductive Behavior of Youth: Pathfinder International’s PRACHAR Project, Bihar, India*. New Delhi, India: Pathfinder International; 2005. Accessed May 19, 2020. http://www2.pathfinder.org/site/DocServer/India-Prachar_Project.pdf

[35] Pathfinder International. *PRAGYA: Multisectoral, Gendered Approach to Improve Family Planning and Sexual and Reproductive Health for Young People: A Research Study*. Watertown, MA: Pathfinder International; 2011. Accessed May 19, 2020. https://www.pathfinder.org/wp-content/uploads/2016/10/PRAGYA-Multisectoral-Gendered-Approach-to-Improve-FP-and-SRH-for-Young-People.pdf

[36] Daniel E, Nanda R. *The Effect of Reproductive Health Communication Interventions on Age at Marriage and First Birth in Rural Bihar, India: A Retrospective Study*. Watertown: Pathfinder International; 2012. Accessed May 19, 2020. https://www.pathfinder.org/wp-content/uploads/2016/11/The-Effect-of-Reproductive-health-Communication-Interventions-on-Age-at-Marriage-and-First-Birth-in-Rural-Bihar-India.pdf

[37] Abuya B, Ngware M, Hungi N, et al. *Community Participation and After-School Support to Improve Learning Outcomes and Transition to Secondary School Among Disadvantaged Girls: A Case of Informal Urban Settlements in Nairobi, Kenya*. Nairobi, Kenya: African Population and Health Research Center; 2014. Accessed May 19, 2020. https://aphrc.org/wp-content/uploads/2019/07/Improving-Learning-Outcomes-Midterm-Report-2014.pdf

[38] Erulkar AS, Ettyang LI, Onoka C, Nyagah FK, Muyonga A. Behavior change evaluation of a culturally consistent reproductive health program for young Kenyans. Int Fam Plan Perspect. 2004;30(2):58–67. 15210404 10.1363/3005804

[39] Austrian K, Muthengi E. *Safe and Smart Savings Products for Vulnerable Adolescent Girls in Kenya and Uganda: Evaluation Report*. Nairobi, Kenya: Population Council; 2013. Accessed May 19, 2020. https://www.popcouncil.org/uploads/pdfs/2013PGY_SafeSmartSavingsEvalReport.pdf

[40] Erulkar A, Chong E. *Evaluation of a Savings & Micro-credit Program for Vulnerable Young Women in Nairobi*. Nairobi, Kenya: Population Council; 2005. Accessed May 19, 2020. https://www.issuelab.org/resources/21090/21090.pdf

[41] Villarruel AM, Zhou Y, Gallegos EC, Ronis DL. Examining long-term effects of Cuídate-a sexual risk reduction program in Mexican youth. Rev Panam Salud Publica. 2010;27(5):345–351. 10.1590/s1020-49892010000500004. 20602068

[42] Georgetown University, Institute of Reproductive Health (IRH). *Utilizing Participatory Data Collection Methods to Evaluate Programs for Very Young Adolescents: An Evaluation of Save the Children’s Choices Curriculum in Siraha, Nepal*. Washington, DC: IRH for the U.S. Agency for International Development; 2011. Accessed May 19, 2020. https://resourcecentre.savethechildren.net/node/5520/pdf/5520.pdf

[43] Lundgren R, Beckman M, Chaurasiya SP, Subhedi B, Kerner B. Whose turn to do the dishes? Transforming gender attitudes and behaviours among very young adolescents in Nepal. Gender Development. 2013;21(1):127–145. 10.1080/13552074.2013.767520

[44] Thurman T, Kidman R, Carton T, Chiroro P. Psychological and behavioral interventions to reduce HIV risk: evidence from a randomized control trial among orphaned and vulnerable adolescents in South Africa. AIDS Care. 2016;28 Suppl 1(sup1):8–15. 10.1080/09540121.2016.1146213. 26886261 PMC4828594

[45] Hallman K, Govender K, Roca E, et al. Siyakha Nentsha, a mentor-led multi-component intervention, enhances social, health and financial assets of rural South African young people. Preprint. Posted online May 28, 2020. Figshare. 10.6084/m9.figshare.12385667.v1

[46] Jewkes R, Nduna M, Levin J, et al. Impact of Stepping Stones on incidence of HIV and HSV-2 and sexual behaviour in rural South Africa: cluster randomised controlled trial. BMJ. 2008;337:a506. 10.1136/bmj.a506. 18687720 PMC2505093

[47] Jewkes R, Nduna M, Levin J, et al. A cluster randomized‐controlled trial to determine the effectiveness of Stepping Stones in preventing HIV infections and promoting safer sexual behaviour amongst youth in the rural Eastern Cape, South Africa: trial design, methods and baseline findings. Trop Med Int Health. 2006;11(1):3–16. 10.1111/j.1365-3156.2005.01530.x. 16398750

[48] Buehren N, Goldstein M, Gulesci S, Sulaiman M, Yam V. *Evaluation of layering microfinance on an adolescent development program for girls in Tanzania*. *Policy Research Working Paper No. 7961*. Washington, DC: World Bank; 2017. Accessed May 19, 2020. http://hdl.handle.net/10986/26025

[49] Hallman K, Mubayiwa R, Madya S, Jenkins A, Goodman S. *Intervention versus Comparison Endline Survey of the Mabinti Tushike Hatamu! (Girls Lets Be Leaders!) Programme in Tanzania*. New York: Population Council, The CSR Group Africa Limited, Restless Development Tanzania, United Nations Children’s Fund Tanzania, Tanzania AIDS Commission; 2016. Accessed May 19, 2020. https://www.unicef.org/tanzania/media/531/file/Tanzania-2016-MTH-survey-report.pdf

[50] Carlson M, Brennan RT, Earls F. Enhancing adolescent self-efficacy and collective efficacy through public engagement around HIV/AIDS competence: a multilevel, cluster randomized-controlled trial. Soc Sci Med. 2012;75(6):1078–1087. 10.1016/j.socscimed.2012.04.035. 22703885

[51] Bandiera O, Buehren N, Burgess R, et al. Women’s empowerment in action: evidence from a randomized control trial in Africa. Am Econ J: Appl Econ. 2020;12(1):210–259. 10.1257/app.20170416

[52] Austrian K, Muthengi E. Can economic assets increase girls’ risk of sexual harassment? Evaluation results from a social, health and economic asset-building intervention for vulnerable adolescent girls in Uganda. Child Youth Serv Rev. 2014;47(2):168–175. 10.1016/j.childyouth.2014.08.012

[53] Nabunya P, Ssewamala FM, Mukasa MN, Byansi W, Nattabi J. Peer mentorship program on HIV/AIDS knowledge, beliefs, and prevention attitudes among orphaned adolescents: an evidence based practice. Vulnerable Child Youth Stud. 2015;10(4):345–356. 27042195 PMC4814228

[54] Jennings L, Ssewamala FM, Nabunya P. Effect of savings-led economic empowerment on HIV preventive practices among orphaned adolescents in rural Uganda: results from the Suubi-Maka randomized experiment. AIDS Care. 2016;28(3):273–282. 10.1080/09540121.2015.1109585. 26548549 PMC4747687

[55] Ssewamala FM, Han C-K, Neilands TB. Asset ownership and health and mental health functioning among AIDS-orphaned adolescents: findings from a randomized clinical trial in rural Uganda. Social Sci Med. 2009;69(2):191–198. 10.1016/j.socscimed.2009.05.019. 19520472 PMC2819297

[56] Ssewamala FM, Ismayilova L. Integrating children’s savings accounts in the care and support of orphaned adolescents in rural Uganda. Soc Serv Rev. 2009;83(3):453–472. 10.1086/605941. 20445763 PMC2863345

[57] Ssewamala FM, Ismayilova L, McKay M, Sperber E, Bannon W Jr, Alicea S. Gender and the effects of an economic empowerment program on attitudes toward sexual risk-taking among AIDS-orphaned adolescent youth in Uganda. J Adolesc Health. 2010;46(4):372–378. 10.1016/j.jadohealth.2009.08.010. 20307827 PMC2844862

[58] Ssewamala FM, Nabunya P, Mukasa NM, Ilic V, Nattabi J. Integrating a mentorship component in programming for care and support of AIDS-orphaned and vulnerable children: lessons from the Suubi and Bridges Programs in sub-Saharan Africa. Glob Soc Welf. 2014;1(1):9–24. 10.1007/s40609-014-0008-7. 24999449 PMC4078881

[59] Pham V, Nguyen H, Tho LH, et al. Evaluation of three adolescent sexual health programs in Ha Noi and Khanh Hoa Province, Vietnam. AIDS Res Treat. 2012;2012:986978. 10.1155/2012/986978. 22666565 PMC3362850

[60] Kaljee LM, Genberg B, Riel R, et al. Effectiveness of a theory-based risk reduction HIV prevention program for rural Vietnamese adolescents. AIDS Educ Prev. 2005;17(3):185–199. 10.1521/aeap.17.4.185.66534. 16006206

[61] Austrian K, Hewett P, Soler-Hampejsek E, Bozzani F, Behrman J, Digitale J. *Adolescent Girls Empowerment Programme: Research and Evaluation Mid-Term Technical Report*. Lusaka, Zambia: Population Council; 2016. Accessed May 19, 2020. https://www.popcouncil.org/uploads/pdfs/2016PGY_AGEPMidtermReport.pdf

[62] Austrian K, Hewett P, Soler-Hampejsek E, Digitale J. *Adolescent Girls Empowerment Program (AGEP): Evaluation–Round 4 update*. Lusaka, Zambia: Population Council; 2017. Accessed May 19, 2020. https://www.popcouncil.org/uploads/pdfs/2017PGY_AGEP-EvalRound4.pdf

[63] Cowan FM, Pascoe SJ, Langhaug LF, et al. The Regai Dzive Shiri Project: results of a randomised trial of an HIV prevention intervention for Zimbabwean youth. AIDS. 2010;24(16):2541–2552. 10.1097/QAD.0b013e32833e77c9. 20881473 PMC3058934

[64] Cowan FM, Pascoe SJ, Langhaug LF, et al. The Regai Dzive Shiri Project: a cluster randomised controlled trial to determine the effectiveness of a multi‐component community‐based HIV prevention intervention for rural youth in Zimbabwe–study design and baseline results. Trop Med Int Health. 2008;13(10):1235–1244. 10.1111/j.1365-3156.2008.02137.x. 18778329

[65] Dunbar MS, Dufour M-SK, Lambdin B, Mudekunye-Mahaka I, Nhamo D, Padian NS. The SHAZ! project: results from a pilot randomized trial of a structural intervention to prevent HIV among adolescent women in Zimbabwe. PLoS One. 2014;9(11):e113621. 10.1371/journal.pone.0113621. 25415455 PMC4240618

[66] Prochaska JO, Velicer WF. The transtheoretical model of health behavior change. Am J Health Promot. 1997;12(1):38–48. 10.4278/0890-1171-12.1.38. 10170434

[67] Taukobong HF, Kincaid MM, Levy JK, et al. Does addressing gender inequalities and empowering women and girls improve health and development programme outcomes? *Health Policy Plann*. 2016;31(10):1492–1514. 10.1093/heapol/czw074 27371549

[68] Glanz K, Rimer BK, Viswanath K, eds. Theory, research, and practice in health behavior and health education. In: *Health Behavior and Health Education: Theory, Research, and Practice*. 4th ed. San Francisco, CA: Jossey-Bass; 2008;3:22–44.

[69] Patton GC, Sawyer SM, Santelli JS, et al. Our future: a Lancet commission on adolescent health and wellbeing. Lancet. 2016;387(10036):2423–2478. 10.1016/s0140-6736(16)00579-1. 27174304 PMC5832967

[70] Hinson L, Clement R, Thompson L. *Voice, Choice and Power: Evidence and Recommendations for Increasing Girls’ and Young Women’s Agency and Decision-Making Through U.S*. *Foreign Assistance*. Washington, DC: International Center for Research on Women; 2019. Accessed May 19, 2020. https://www.icrw.org/wp-content/uploads/2019/08/Voice-Choice-and-Power.pdf

[71] Moher D, Hopewell S, Schulz KF, et al. CONSORT 2010 explanation and elaboration: updated guidelines for reporting parallel group randomised trials. Int J Surg. 2012;10(1):28–55. 10.1016/j.ijsu.2011.10.001. 22036893

[72] Des Jarlais DC, Lyles C, Crepaz N, TREND Group. Improving the reporting quality of nonrandomized evaluations of behavioral and public health interventions: the TREND statement. Am J Public Health. 2004;94(3):361–366. 10.2105/ajph.94.3.361. 14998794 PMC1448256

